# A laparoscopic technique for excision of retropubic midurethral sling arms eroding into the bladder

**DOI:** 10.1007/s00192-019-03886-0

**Published:** 2019-02-19

**Authors:** Emily Claire Carter, Rufus Cartwright, Elizabeth Goodall, Simon Jackson, Natalia Price

**Affiliations:** John Radcliffe Hospital, Oxford University Hospitals NHS Foundation Trust, Headley Way, Headington, Oxford, OX3 9DU UK

**Keywords:** Midurethral sling, Laparoscopy, Complications

## Introduction

Midurethral polypropylene slings have a range of rare but serious complications, including pain, recurrent infection, and lower urinary tract erosion. Bladder erosions may be asymptomatic but frequently present with any combination of vaginal symptoms (pain or dyspareunia), storage symptoms (recurrent incontinence or urinary urgency), voiding symptoms, and recurrent urinary tract infection (rUTI) [1]. Management involves partial or complete excision of the sling, with different risks associated with each.

Previous reports have described cystoscopic approaches with mechanical trimming or holmium-YAG pulsed ablation of the visible mesh [2] and vesicoscopic approaches with incision at the bladder dome [3, 4]. These approaches provide good exposure for excision of eroded intramucosal portions of mesh; however, intradetrusor mesh is left in situ with a subsequent risk of recurrent mesh erosion. Robotic assistance in complex laparoscopic surgery is becoming increasingly used due to improved dexterity, depth perception, and absence of fatigue; however, more widespread use is limited currently by its cost.

Open surgery with cystotomy at the dome allows complete mesh excision from the bladder wall, including intramural and intradetrusor portions; however, open surgery has associated risks and longer recovery. The laparoscopic technique described here has advantages, including precision of dissection, complete excision of mesh from the bladder wall, and rapid recovery.

We demonstrate in this video a laparoscopic technique for excision of midurethral polypropylene slings eroding into the bladder that can be used for excision of an eroding sling from the intramucosal and intradetrusor portions of the bladder wall. This can be reproduced by experienced laparoscopic surgeons by following our step-by step technique.

## Method

**Fig. 1:**
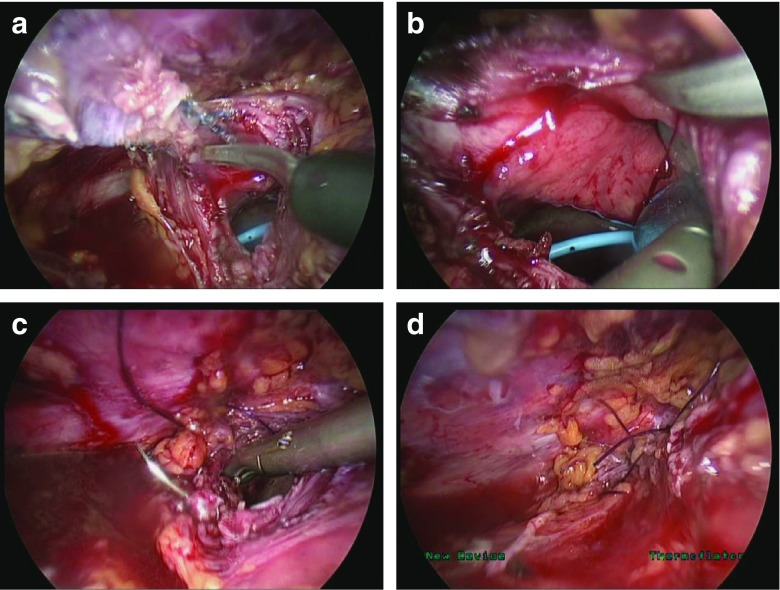
Laparoscopic excision of mesh eroding into the bladder.** a** a cystotomy is made where the tape breaches the bladder wall,** b** the tape is completely freed from the bladder using scissors,** c** the cystotomy is sutured in two layers with polyglactin suture,** d** a check for water-tight closure is made

All surgeries were completed at one urogynecology unit by one of two experienced laparoscopic urogynecologists (SRJ or NP). After careful preoperative patient counselling and placement of ureteric stents, if required, the bladder is instilled with 300ml of normal saline with methylene blue to help delineate the dome of the bladder. With the patient in Trendelenburg position, the retropubic space is opened using a monopolar hook 2 cm above the bladder reflection. The space of Retzius is developed, and the bladder is carefully reflected to demonstrate the urethra and sphincter complex in the midline and the obturator vessels and nerves bilaterally (Fig. 1). The arms of the mesh are identified, and the relationship of the mesh to the important structures in the retropubic space is assessed. Under traction, the mesh is sharply dissected out from the surrounding structures. This is continued down to the level of the bladder. The cystotomy is made at the point(s) where the sling erodes into the bladder. A mesh excision is performed, whereby the eroded sling arm(s) are carefully removed laparoscopically from the bladder. The intradetrusor and intramucosal portions of the mesh are completely removed to prevent further erosion. The suburethral portion remains in situ to reduce the risk of recurrence of stress incontinence. The cystotomy is closed in two layers using a polyglactin suture. An indwelling catheter is left for 2 weeks to allow bladder healing, with a cystogram performed prior to catheter removal.

## Results

We identified six women undergoing this procedure at a median 45 months after midurethral sling (MUS) insertion. Two of these women had a history of prior mesh removal surgery involving cystoscopic excision or ablation of intramucosal mesh. These two patients developed recurrence of mesh erosion from the intraluminal portion of the mesh and were referred to our center for a second mesh excision. The procedures took a median 112 min (excluding one patient with bilateral erosion and bilateral cystotomy: 240 min). There were no early complications, and no cases returned to theater. We followed up all patients with a questionnaire a minimum of 24 months postsurgery. For the four patients with preoperative pain associated with the mesh, there was a median 7-point decrease in pain (10-point scale). Two patients had subsequent surgery under our care to remove suburethral mesh due to pain after the index surgery. There were no recurrences of mesh erosion into the bladder after excision, and no cases of fistula formation.

## Discussion

Advantages of the laparoscopic approach include magnified visualization of anatomical structures with more precise complete excision of eroded intramural and intradetrusor portions of mesh, minimizing the recurrence risk of mesh erosion. Patients additionally benefit from reduced postoperative pain, shorter admission time, and a quicker return to activities of daily living.

A combined laparoscopic and vaginal approach can aid in releasing the suburethral portion of the sling, but in the setting of a bladder erosion, a vaginal incision has a risk of creating a vesicovaginal fistula, necessitating use of an interposition flap. Complete removal in one stage is not always appropriate in all cases, and specifically, if the mesh has eroded in close proximity to the bladder neck or urethra, there is a higher risk of fistula formation. If complete excision is desired, a planned two-stage procedure could be a safer option, minimiszing the risk of fistula formation. Due to preservation of the suburethral portion of the mesh in patients in this case series, incontinence was not a major issue at routine postoperative follow-up.

## Conclusion

Our series confirms the feasibility of this technique [5] with some advantages over cystoscopic or open approaches. These include precise dissection under direct vision, which gives better exposure and identification of anatomical structures, and the opportunity for a complete excision of intramucosal and intradetrusor portions of the mesh to prevent recurrence, which is not possible with cystoscopic or vesioscopic approaches. The surgery is less complicated, with a quicker recovery and better cosmetic approach when compared with the open approach. We therefore recommend the laparoscopic approach for removal of bladder-mesh erosion, and our technique can be reproduced by experienced laparoscopic surgeons.

## Electronic supplementary material

ESM 1 (MP4 51,010 kb)
